# Phosphoproteomic Analysis of *Xenopus laevis* Reveals Expression and Phosphorylation of Hypoxia-Inducible PFKFB3 during Dehydration

**DOI:** 10.1016/j.isci.2020.101598

**Published:** 2020-09-22

**Authors:** Liam J. Hawkins, Xiaoshuang Wang, Xiaomin Xue, Hui Wang, Kenneth B. Storey

**Affiliations:** 1 Department of Biology, Carleton University, 1125 Colonel By Drive, Ottawa, ON K1S 5B6, Canada; 2 Key Laboratory of Animal Physiology, Biochemistry and Molecular Biology of Hebei Province, College of Life Sciences, Hebei Normal University, Shijiazhuang, Hebei 050024, China

**Keywords:** Animal Physiology, Cell Biology, Proteomics

## Abstract

*Xenopus laevis* tolerate dehydration when their environments evaporate during summer months. Protein phosphorylation has previously shown to regulate important adaptations in *X. laevis*, including the transition to anaerobic metabolism. We therefore performed phosphoproteomic analysis of *X. laevis* to further elucidate the cellular and metabolic responses to dehydration. Phosphoproteins were enriched in cellular functions and pathways related to glycolysis/gluconeogenesis, the TCA cycle, and protein metabolism, among others. The prominence of phosphoproteins related to glucose metabolism led us to discover that the hypoxia-inducible PFKFB3 enzyme was highly phosphorylated and likely activated during dehydration, a feature of many cancers. Expression of the four transcript variants of the *pfkfb3* gene was found all to be upregulated during dehydration, potentially due to the enrichment of hypoxia responsive elements in the *pfkfb3* promoter. These results further support the role of anaerobic glycolysis during dehydration in *X. laevis* and elucidate a potential mechanism for its increased rate.

## Highlights

• Clustering analysis indicated a concerted response in liver but not skeletal muscle
• Phosphoproteins were enriched for glycolysis, gluconeogenesis, and the TCA cycle
• Phosphopeptides of hypoxia-inducible PFKFB3 were highly abundant during dehydration
• Four PFKFB3 variants were upregulated potentially supporting anaerobic glycolysis

## Graphical Abstract

**Figure:**
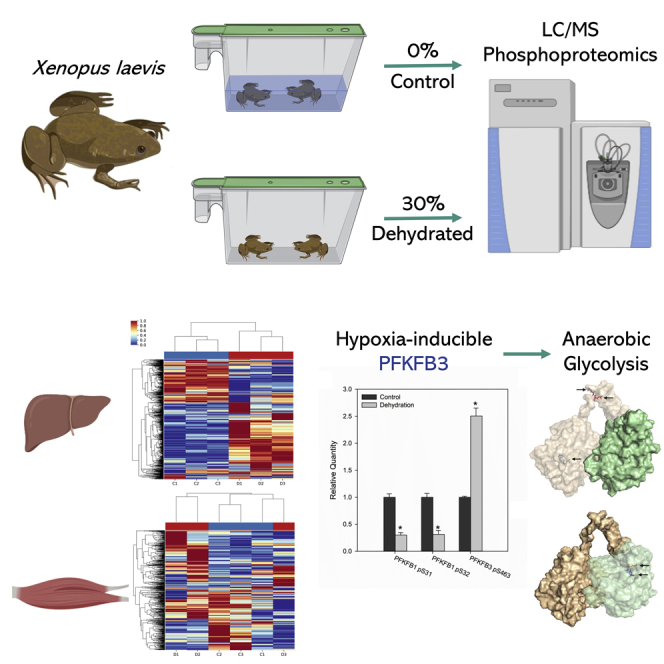

## Introduction

The African clawed frog (*Xenopus laevis*) is one of a select group of amphibians that encounters and tolerates severe dehydration in their native habitats. When the bodies of water they occupy dry up during the summer months, these mostly aquatic animals are exposed to evaporative dehydration and can survive beyond 30% body water loss (Hillman, 1978a). Interestingly, although *X. laevis* have been intensely characterized within the context of their role as model organisms for developmental and cellular biology, their adaptations necessary for surviving dehydration are understudied.

It is known that the response to dehydration in *X. laevis* manifests in a tissue-specific manner. At an organismal level, blood is preferentially distributed during dehydration such that skeletal muscle has the greatest loss, whereas blood distribution to the brain actually increases (Hillman and Sommerfeldt, 1981). This results in disparate levels of dehydration stress that individual tissues have adapted to by harboring different functions in response to dehydration. For example, the skin secretes a mucous layer to minimize evaporative dehydration, the heart sustains a high rate to maximize oxygen delivery (Hillman, 1978a), and the liver switches from ammonotelism to ureotelism to avoid a toxic buildup of nitrogenous waste (Balinsky et al., 1961). At high levels of dehydration, *X. laevis* increase rates of anaerobic glycolysis as apparent by elevated whole-body lactate levels (Hillman, 1978a).

Investigation into metabolic adaptations has focused on glucose metabolism due to the dependency on anaerobic glycolysis, particularly in the liver. In this tissue, enzymology studies have indicated that pyruvate kinase (PK) has a greater activity during dehydration than under control conditions (Dawson et al., 2018). This is of importance since it is an ATP-producing step in glycolysis and generally considered to be a rate-limiting enzyme under tight regulation in the liver. Increased activity of PK in the liver would support the increased rate of anaerobic glycolysis, which is further evident by a study examining lactate dehydrogenase (LDH). LDH in this animal was shown to have decreased affinity for its substrates, although this affinity is restored in the presence of urea concentrations seen during dehydration (Katzenback et al., 2014). In skeletal muscle, hexokinase also has altered kinetics (Childers and Storey, 2016) that may regulate glucose entry into muscle cells when plasma glucose levels are elevated during dehydration (Malik and Storey, 2009).

The commonality between the above-mentioned glycolytic enzyme studies is the observed changes in phosphorylation state that provide a mechanism to explain their altered kinetics during dehydration. Phosphorylation is a quick and reversible method of altering protein function and is a known regulator of metabolic pathways and cellular processes during environmental stress responses (Cowan and Storey, 2003; Storey, 2002; Storey and Storey, 1990). By examining changes in phosphorylation patterns, we can determine which pathways and processes are being actively regulated in response to a variety of environmental stresses and broaden our understanding of adaptations necessary for tolerating them. This has been explored in a variety of species including hibernating mammals (Chung et al., 2013; Vertommen et al., 2017) and most recently in the freeze-, anoxia-, and dehydration-tolerant frog species, *Rana sylvatica* (Hawkins et al., 2019).

Like *X. laevis*, *R. sylvatica* is tolerant to extreme levels of body water loss (Churchill and Storey, 1994). Phosphoproteomic analysis indicates that several enzymes involved in glycogenolysis and glycolysis are differentially phosphorylated and may function to mobilize glycogen stores to supply fuel for anaerobic glycolysis during dehydration (Hawkins et al., 2019). This analysis proved fruitful for expanding what we know about how this anuran can tolerate multiple stresses.

Here we take a similar approach to analyze changes in the phosphoproteome of *X. laevis* in response to dehydration exposure. We exposed adult *X. laevis* to dehydrating conditions and used liquid chromatography-tandem mass spectrometry (LC-MS/MS) to quantify phosphopeptides from the liver and skeletal muscle of these animals. This is followed by bioinformatic analysis to gain an overview of the processes and pathways responding to dehydration and broaden our understanding of dehydration tolerance.

## Results

### Phosphoproteomics Summary

Phosphoproteomic analysis resulted in the identification of 787 unique phosphopeptides in the liver and 692 in skeletal muscle, which matched to 824 and 396 proteins, respectively, including proteins with shared peptides. Both liver and skeletal muscle showed similar proportions of phosphorylated residues (Figure S1A). In the liver, there were 715 (82.66%) phosphoserine residues, 130 (15.03%) phosphothreonine residues, and 20 (2.31%) phosphotyrosine residues. In skeletal muscle, there were 633 (80.74%) phosphoserine, 134 (17.09%) phosphothreonine, and 17 (2.17%) phosphotyrosine residues. The number of phosphorylated residues per phosphopeptide was also comparable between tissues (Figure S1B). In the liver, 711 (90.34%) phosphopeptides had one phosphoresidue, 74 (9.40%) had two phosphoresidues, and 2 (0.25%) had three or more phosphoresidues. In muscle, 608 (87.86%) phosphopeptides had one phosphoresidue, 76 (10.98%) had two phosphoresidues, and 8 (1.16%) had three or more phosphoresidues.

### Dehydration Produces Distinct Phosphorylation Patterns

**Figure 1:**
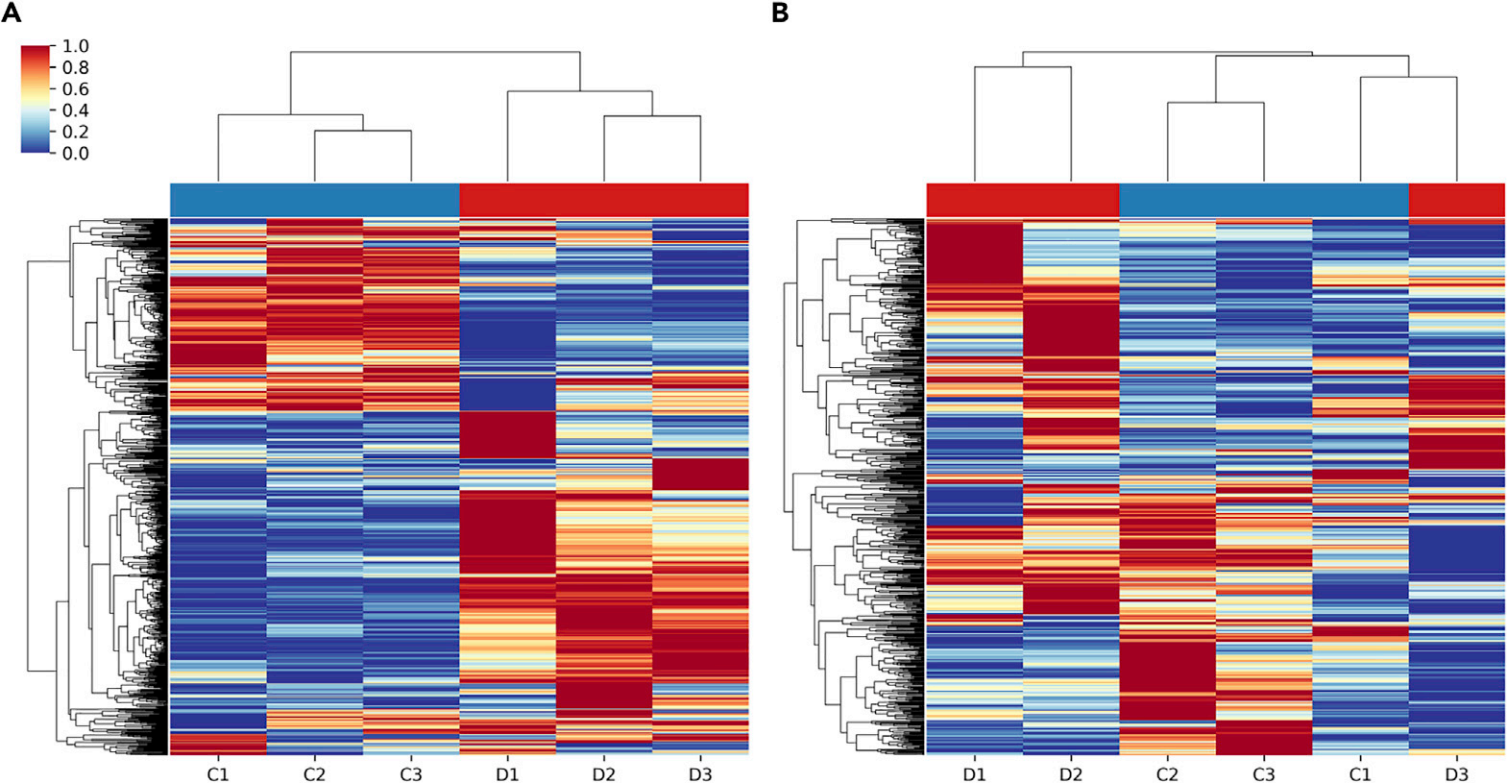
Hierarchical Clustering Analysis of Phosphopeptides in the Liver and Muscle of *Xenopus Laevis* in Response to Dehydration Clustering was performed on samples from control and dehydrated animals in (A) liver and (B) skeletal muscle. Samples (arranged horizontally) were clustered by Euclidean distance between relative quantities of phosphopeptides, and phosphopeptides (arranged vertically) were clustered based on Euclidean distance between each sample. Colors indicate *Z* score (standardized quantities) where red is a high *Z* score and blue is a low *Z* score. The experimental group the sample belongs to is indicated by blue (control) or red (dehydrated) bars above the heatmap.

Hierarchical clustering of relative quantities of all phosphopeptides resulted in clustering of control samples together and dehydration samples together in liver (Figure 1A). This contrasted clustering in muscle, which showed intermixing of control and dehydration samples when using all phosphopeptides (Figure 1B), indicated less of a defined phosphorylation pattern differentiating control and dehydrated animals in this tissue. Unsurprisingly, when hierarchical clustering was performed on only statistically differentially phosphorylated peptides both tissues showed clear clustering of samples from control and dehydrated animals (Figure S2).

**Figure 2:**
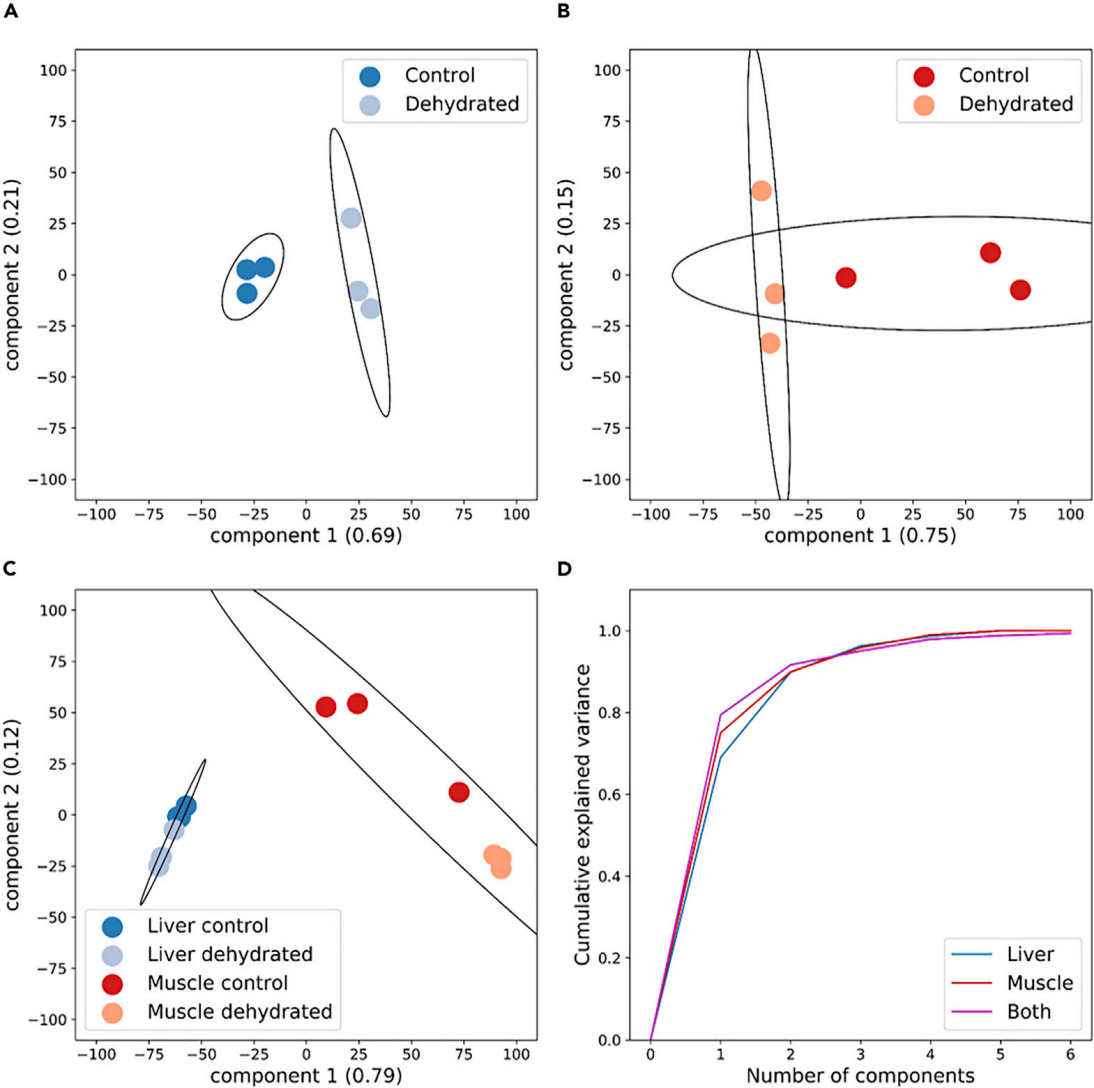
Principal Component Analysis of Samples in the Liver and Muscle of *Xenopus Laevis* in Response to Dehydration Principle component analysis was performed on phosphopeptide relative quantities in (A) liver, (B) skeletal muscle, and (C) both tissues. The cumulative explained variance of principal components is plotted in (D).

PCA resulted in similar conclusions to hierarchical clustering results. In liver, samples from control and dehydrated animals clustered together and were particularly separable along the first component (explaining 69% of the variance between samples) (Figure 2A), whereas there was less separation and clustering of control and dehydrated muscle samples (Figure 2B). These results again support a stronger distinct phosphorylation pattern in the liver than in muscle. When analyzed together, PCA analysis showed higher inter-tissue separation than separation between experimental conditions (Figure 2C), although this is expected given the dissimilarity between liver and muscle tissue. It should be noted that, when PCA analysis was performed on both tissues together, only the subset of phosphopeptides that appeared in both tissues could be examined (125 phosphopeptides), versus a much higher number that appeared in each tissue individually (787 in liver, 692 in muscle). When PCA was performed on tissues separately and together, cumulative explained variance curves were similar (Figure 2D).

### Liver Phosphopeptides Are Differentially Abundant

**Figure 3:**
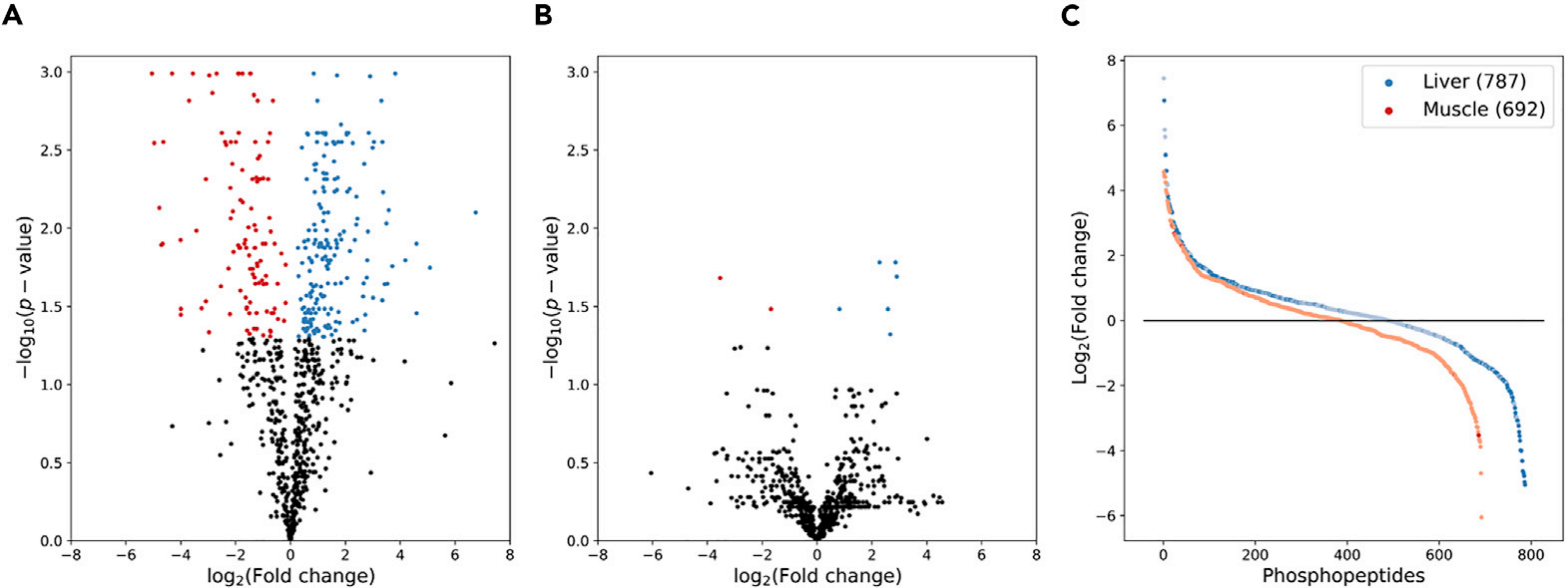
Volcano Plot of Phosphopeptides from Liver and Muscle of *Xenopus Laevis* in Response to Dehydration Phosphopeptides that had significantly increased (FDR-corrected p value < 0.05) and decreased relative abundance in dehydration samples are in blue and red, respectively, for (A) liver and (B) skeletal muscle. Phosphopeptides from liver and skeletal muscle ordered by Log_2_ fold-change is shown in (C).

Significant differences in relative phosphopeptide amounts varied between the two tissues to a much greater extent than would be predicted by the number of phosphopeptides found in each tissue (Figure 3). In the liver, 308 (39.14%) phosphopeptides had false discovery rate (FDR)-corrected p values < 0.05 (Figure 3A), whereas in muscle only 8 (1.2%) phosphopeptides met this criteria (Figure 3B). This discrepancy appears to be due to intra-experimental condition variable since the number and distribution of phosphopeptides with high and low fold-changes are comparable between liver and muscle (Figure 3C). These results further support the distinct phosphorylation patterns present in the liver and not muscle as seen in the clustering analysis.

**Figure 4:**
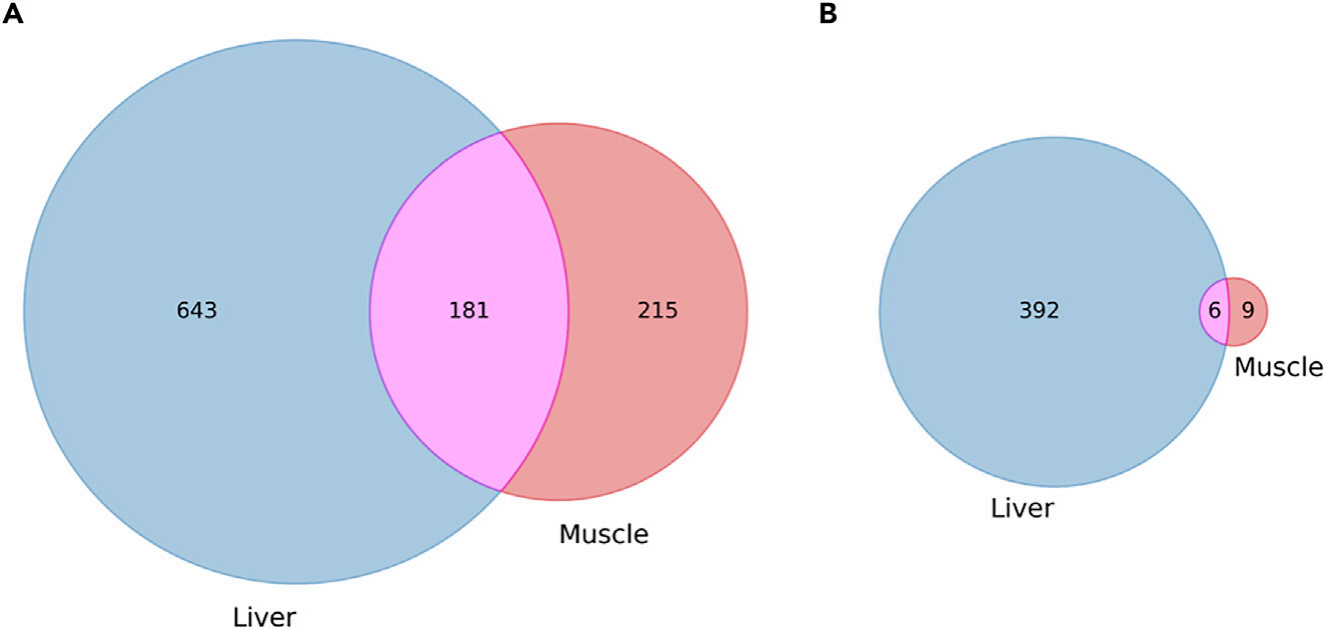
Venn Diagram of Phosphoproteins from Liver and Muscle of *Xenopus Laevis* in Response to Dehydration Phosphoproteins with at least one corresponding phosphopeptide identified in liver and/or muscle is shown in (A), whereas only those with at least one significantly differentially abundant phosphopeptides are shown in (B).

By comparing proteins with corresponding phosphopeptides found in each tissue, the overlap of proteins is substantial where 181 proteins are found in common (21.97% of liver proteins, 45.71% of muscle proteins) (Figure 4A). When only proteins with corresponding significantly differentially abundant phosphopeptides are considered this number drops to six proteins (1.51% of liver proteins, 40.00% of muscle proteins) (Figure 4B), indicating that the vast majority of affected proteins in the liver are liver specific, whereas the majority of affected proteins in the muscle are not muscle specific but are also affected in the liver.

### Overrepresentation of GO Terms and KEGG Pathways

**Figure 5:**
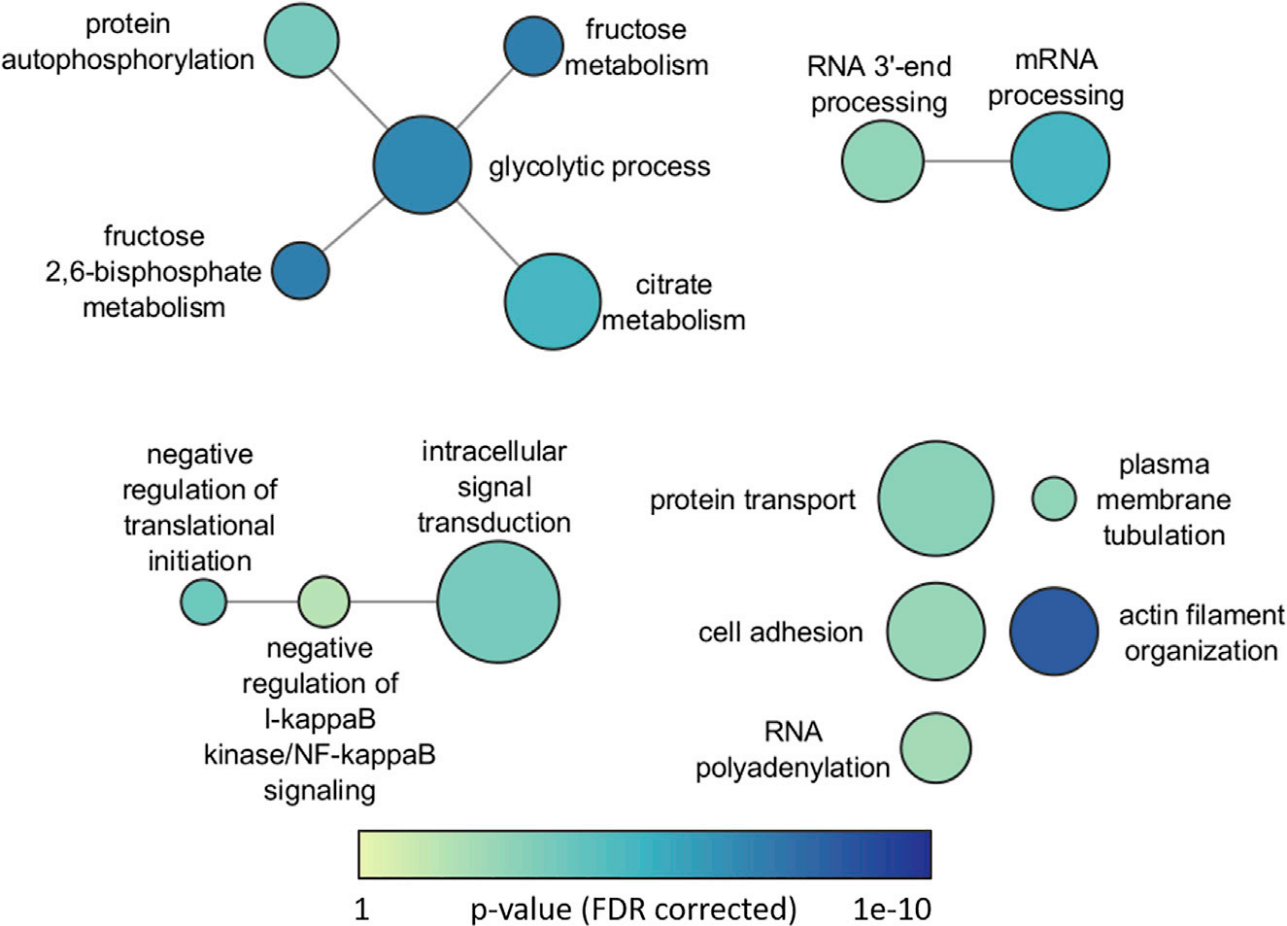
Enriched Gene Ontology Biological Processes Semantic Relation Network in the Liver of *Xenopus Laevis* Exposed to Dehydration Enriched GO biological processes terms were summarized by REVIGO (Supek et al., 2011) and resulting semantic relation network was plotted using Cytoscape. Node size is proportional to the number of genes each term encompasses and the darker the blue the lower the FDR-adjusted p value. Edges indicate semantic relationship.

REVIGO analysis revealed enrichment of differentially abundant phosphopeptides in small networks of semantically related non-redundant GO biological processes in the liver (Figure 5). These include a glucose metabolism-related network (glycolytic process, fructose metabolism, citrate metabolism, fructose 2,6-bisphosphate metabolism, and protein autophosphorylation), a post-transcriptional regulation network (negative regulation of translational initiation, negative regulation of IκB kinase/NF-κB signaling, and intracellular signal transduction), and an RNA processing network (RNA 3′-end processing and mRNA processing), along with other unconnected nodes (protein transport, plasma membrane tubulation, cell adhesion, actin filament organization, and RNA polyadenylation). GO cellular compartments were also enriched, namely, microtubule and cytoskeletal compartments (Figure S3), whereas enriched GO molecular functions included an actin related network and a kinase related network (Figure S4).

**Figure 6:**
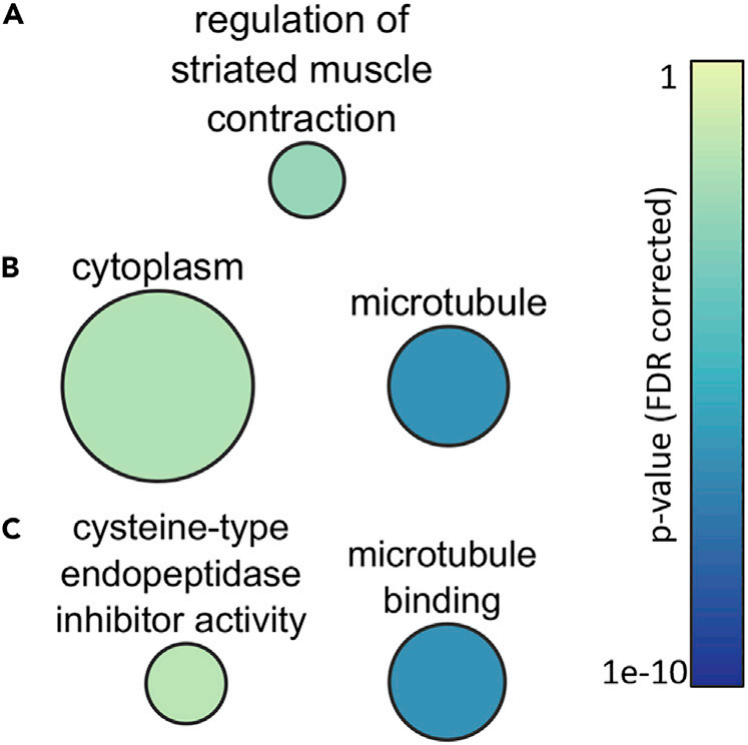
Enriched Gene Ontology Term Semantic Relation Networks in Skeletal Muscle of *Xenopus Laevis* Exposed to Dehydration GO biological processes are in (A), cellular compartments in (B), and molecular functions in (C). All other details are as in Figure 5.

REVIGO analysis of muscle samples showed few enriched GO terms given the low number of differentially abundant phosphopeptides (Figure 6). Only a single GO biological process was enriched, regulation of striated muscle contraction (Figure 6A), two GO cellular compartments, cytoplasm and microtubule (Figure 6B), and two GO molecular functions, cysteine-type endopeptidase inhibitor activity and microtubule binding (Figure 6C).

**Figure 7:**
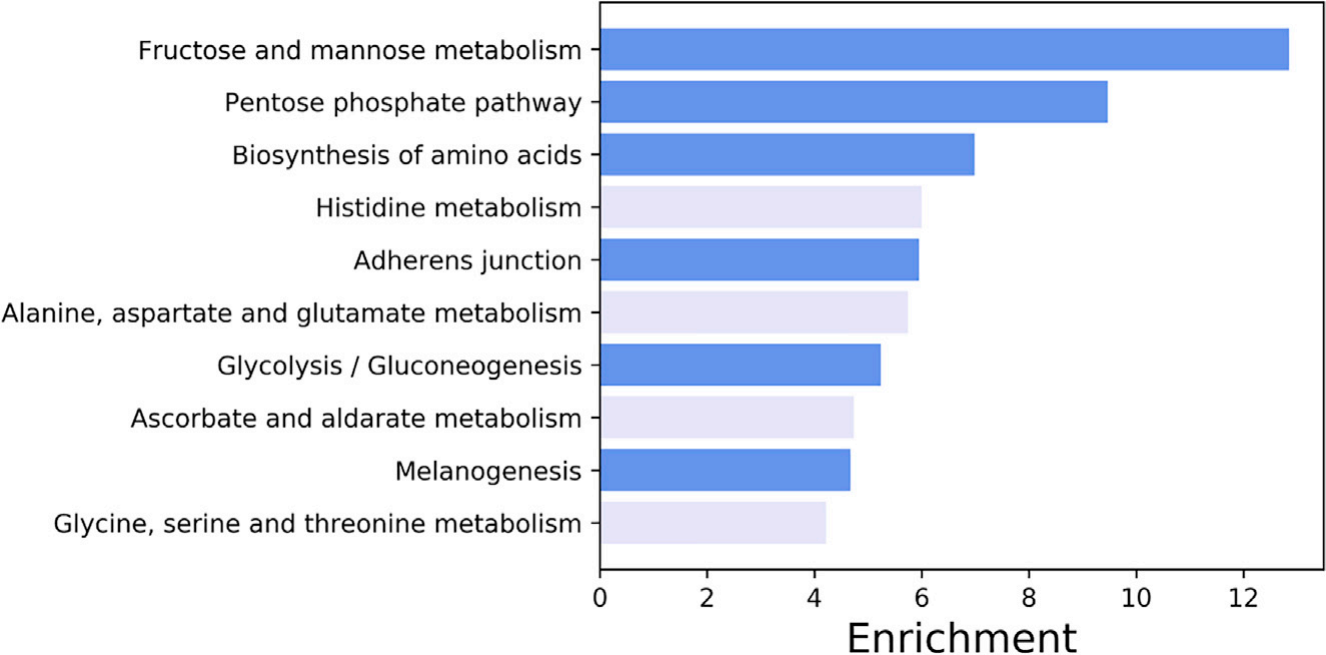
Top Ten Enriched Kyoto Encyclopedia of Genes and Genomes Pathways in the Liver of *Xenopus Laevis* Exposed to Dehydration Terms in dark blue are statistically enriched (FDR-corrected p value < 0.05), terms in light blue are not significantly enriched.

KEGG pathways enriched for proteins with differentially abundant phosphopeptides in the liver pertained mainly to central metabolic pathways: fructose and mannose metabolism, pentose phosphate pathway, biosynthesis of amino acids, and glycolysis/gluconeogenesis, and also adherens junction and melanogenesis (Figure 7). No KEGG pathways were significantly enriched in the muscle.

### PFKFB3 Is Phosphorylated and Upregulated during Dehydration

Seeing that central carbon pathways were enriched for proteins corresponding to differentially abundant phosphopeptides in the liver, we investigated which enzymes were contributing to this enrichment (Table 1). Two enzymes related to glycogen metabolism, glycogen synthase kinase-3 beta (GSK3β), and phosphoglucomutase (PGM) had increased phosphopeptide abundance, whereas only one TCA cycle enzyme, ATP-citrate synthase (CS), showed significant differences in phosphopeptide abundance. Enzymes comprising glycolysis and gluconeogenesis, 6-phosphofructo-2-kinase/fructose-2,6-biphosphatase 1 and 3 (PFKFB1 and PFKFB3), the liver isoform of fructose-bisphosphate aldolase (ALDOB), and glyceraldehyde-3-phosphate dehydrogenase (GAPDH), did, however, all show large changes in phosphopeptide abundance both in magnitude and statistical significance (Table 1).

**Table 1:** Glucose Metabolism-Related Enzymes Corresponding to Identified Phosphopeptides

Pathway	Abbreviation	UniProt	Peptide	Residue	Log2 Fold Change	FDR-Corrected p Value
Glycogen metabolism	GSK3β	Q91757	GEPNV**pS**YICSR	S215	0.67	**0.033**
			GEPNVS**pY**ICSR	Y216	0.59	0.063
	PGM	A0A1L8GFG5	AIGGIIL**pT**ASHNPGGPNGDFGIK	T115	1.20	**0.005**
			AIGGIILTA**pS**HNPGGPNGDFGIK	S117	1.20	**0.009**
Glycolysis/Gluconeogenesis	PFKFB1	Q5U4Z6	RG**pS**SIPQFTNSPTMIIMVGLPAR	S31	−1.74	**0.007**
			RGS**pS**IPQFTNSPTMIIMVGLPAR	S32	−1.67	**0.013**
	PFKFB3	A0A1L8GTR7	RN**pS**VTPLASPEPTKK	S463	1.32	**0.005**
	ALDOB	Q5XHC6	GILAADE**pS**VGTMGSR	S36	−2.17	**0.003**
	GAPDH	P51469	VINDNFGIVEGLMTTVHAFTA**pT**QK	T182	−2.34	**0.003**
	PK	A0A1L8FDJ9	RI**pS**ENMAQLMQDLGPAFVQR	S89	−0.44	0.267
TCA cycle	PDHA1	Q66JA7	YGMGT**pS**VER	S242	−1.19	0.101
			YHGH**pS**MSDPGVSYR	S303	−0.28	0.485
			YHGHSM**pS**DPGVSYR	S305	−0.11	0.785
			YHGHSMSDPGV**pS**YR	S310	1.02	0.186
			YHGHSM**pS**DPGV**pS**YR	S305 + S310	−0.49	0.242
	CS	Q5U5A8	TA**pS**FSESRTEDITPAKK	S455	−1.23	**0.044**

Glycogen metabolism, glycolysis, gluconeogenesis, and TCA cycle enzymes with identified phosphopeptides are presented. FDR corrected p values in bold are statistically significant (p < 0.05).

Abbreviations: GSK3β, glycogen synthase kinase-3 beta; PGM, phosphoglucomutase; PFKFB1, 6-phosphofructo-2-kinase/fructose-2,6-biphosphatase 1; PFKFB3, 6-phosphofructo-2-kinase/fructose-2,6-biphosphatase 3; ALDOB, fructose-bisphosphate aldolase (liver); GAPDH, glyceraldehyde-3-phosphate dehydrogenase; PK, pyruvate kinase; PDHA1, pyruvate dehydrogenase E1 component subunit alpha; CS, ATP-citrate synthase.

**Figure 8:**
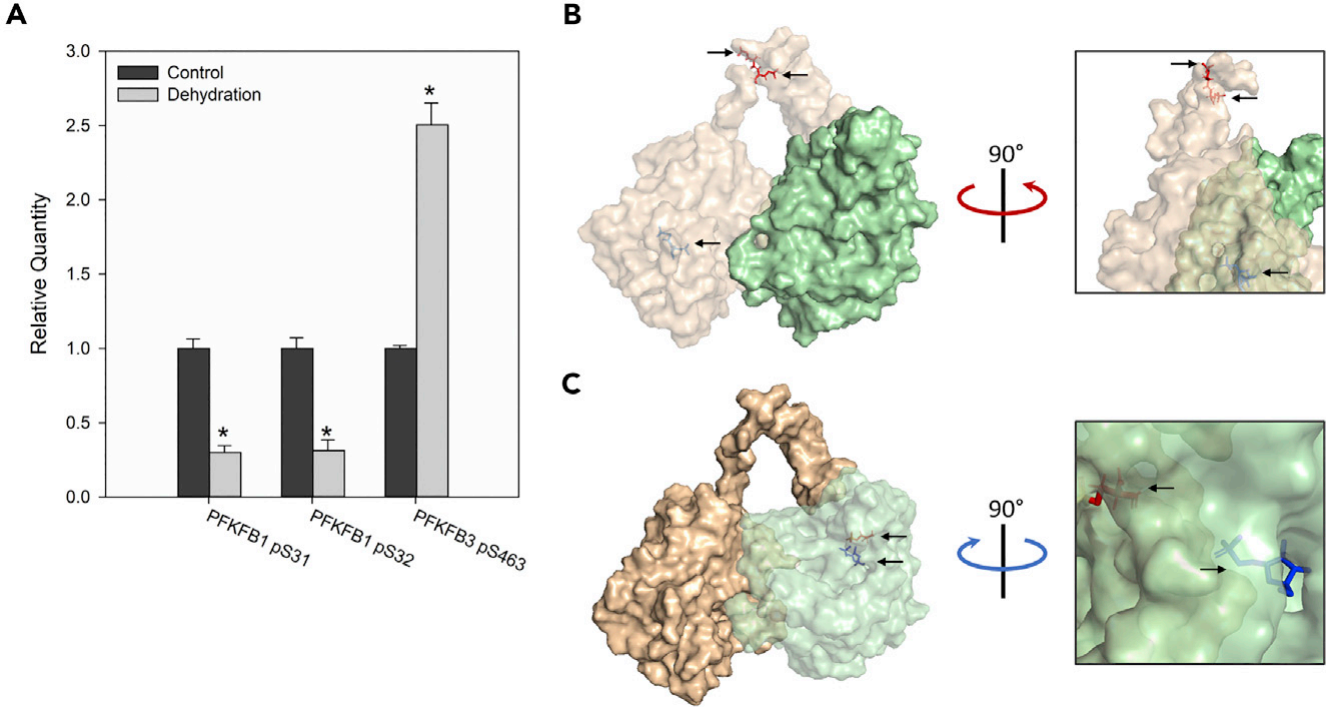
Phosphorylation of Liver PFKFB Enzymes in Response to Dehydration PFKFB1 S31, PFKFB1 S32, and PFKFB3 S463 were identified as differentially phosphorylated in the liver during dehydration and are shown in (A), data are represented as mean ± SEM and ∗ indicates statistical significance (FDR-corrected p value < 0.05) determined by Student's t test. S31 and S32 are present in the PFK2 domain (translucent orange) of PFKFB1 as shown in (B). S463 is present in the FBP2 domain (translucent green) of PFKFB3 as shown in (C). Arrows in (B) and (C) show phosphorylated serines (red) and substrates (blue).

Of particular interest is the phosphorylation pattern of PFKFB1 and 3 in the liver, the bifunctional enzymes that catalyze the interconversion of fructose 6-phosphate (Fru-6-P) and fructose 2,6-bisphosphate (Fru-2,6-P_2_), a potent phosphofructokinase 1 (PFK1) activator (Figure 8A). PFKFB1 is the liver isoform and showed significantly decreased amount of phosphopeptide abundance at S31 and S32 (homologous to S33 and S34 of human PFKFB1). S31 is a known protein kinase A target within the phosphofructokinase-2 (PFK2) domain (Figure 8B), and when phosphorylated it suppresses PFK2 activity and activates fructose-2,6-bisphosphatase (FBP2) activity (Ros and Schulze, 2013). Analogous results are seen with the increased phosphopeptide abundance of PFKFB3 at S463 (homologous to S461 of human PFKFB3) (Figure 8A), which is part of the FBP2 domain that promotes PFK2 activity when phosphorylated (Bando et al., 2005) (Figure 8C). Phosphorylation and activation of PFKFB3 has been shown in a variety of conditions, notably by AMPK under hypoxic conditions (Almeida et al., 2004; Marsin et al., 2002).

**Figure 9:**
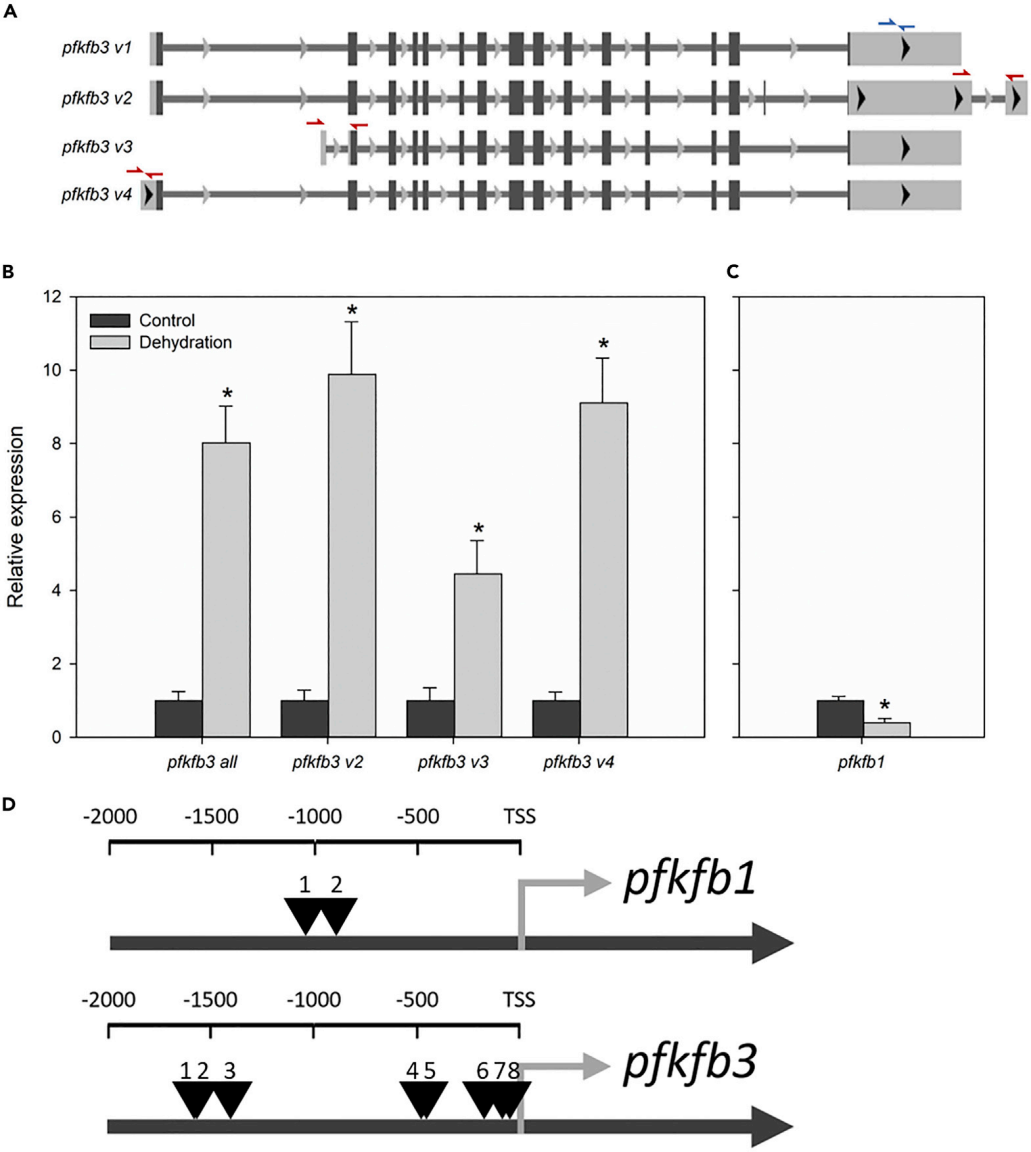
Expression of *pfkfb3* and *pfkfb1* in the Liver during Dehydration Primers were designed to measure the expression of *pfkfb3* transcript variants as shown by colored arrows in (A). Blue arrows show the position of forward and reverse primers for measuring all four *pfkfb3* transcript variants, whereas red arrows show primer pairs for measuring specific variants. Primer sequences are in Table S1. (B) shows relative expression of *pfkfb3* transcript variants in the liver of control and dehydrated *X. laevis*, whereas relative expression of *pfkfb1* is in (C), data are represented as mean ± SEM and ∗ indicates statistical significance (p value < 0.05) determined by Student's t test. (D) shows the position of hypoxia responsive elements (HREs, binding sites for HIF1α) within 2 kbp upstream of the transcription start sites (TSS) of *pfkfb1* and *pfkfb3*.

Expression of PFKFB3 is not typical of liver tissue; however, this isoform is also known as inducible PFKFB, as it has been observed to be expressed in response to hypoxic conditions of cancer cells by hypoxia inducible factor 1-alpha (HIF1α) (Bando et al., 2005; Shi et al., 2017). This isoform has four transcript variants in *X. laevis* (Figure 9A), all of which code for proteins containing the regulatory S463 residue. We designed primers to measure the expression of variants 2, 3, and 4 by RT-qPCR, as well as a primer pair that would amplify all four variants (*pfkfb3 all*), and therefore give some measure incorporating variant 1 expression levels, which otherwise has 100% identity with variants 2 and 4. Expression of variants 2, 3, and 4 increased 9.88 ± 1.43, 4.44 ± 0.91, and 9.11 ± 1.22-fold during dehydration, whereas the primer pair measuring all four variants increased 8.02 ± 1.00-fold relative to control samples (Figure 9B). Increased expression of PFKFB enzymes is not a general response as seen by relative expression levels of PFKFB1, which actually decreased during dehydration (Figure 9B). These results may be explained by the presence of eight hypoxia-responsive elements (HREs, RCGTG [Mole et al., 2009]) within 2 kbp of the *pfkfb3* transcription start site (TSS) (five within ∼500 bp), whereas only two are observed 2 kbp upstream of the *pfkfb1* TSS (Figure 9C).

## Discussion

### Consistent Phosphorylation Pattern in Liver but Not Skeletal Muscle

Our analysis of dehydration exposure in *X. laevis* identified a distinct dehydration-responsive phosphorylation pattern in liver but not in skeletal muscle. These results are consistent with this animal's strategy of dehydration tolerance. In response to dehydration, the liver undergoes relatively large changes to its physiology and metabolism including converting from ammonotelism to ureotelism (Balinsky et al., 1967) and switching to anaerobic glycolysis as oxygen transport is hampered (Katzenback et al., 2014). Both processes are under the control of enzymes that can be phosphorylated to control their activity and kinetics, and thus our detection of dehydration-responsive phosphorylation patterns in this organ was expected. This is reflected in our clustering analysis where samples from control animals clustered separately from dehydration animals (Figure 1A). Likewise, distinct clustering of samples from control and dehydration animals was present in our PCA, where there was no overlap of confidence ellipses representing three standard deviations of the underlying Gaussian distribution (Figure 2A). In contrast to the liver, since these animals assume a water-conserving posture to avoid unnecessary energy expenditure and evaporative dehydration, the muscles are dormant until rehydration occurs. This results in a less defined phosphorylation pattern in muscle relative to liver, where samples did not cluster with their experimental conditions (Figure 1B). Similarly, PCA of muscle samples showed much greater intra-condition variability and overlap of underlying distributions (Figure 2B). The overall difference between the tissues and their response to dehydration is seen when PCA was performed on all samples together. Although this was limited to the small subset of phosphopeptides present in both tissues, it is clear that there are greater differences at this level between tissues than between experimental conditions within each tissue (Figure 2C). Again, this difference between tissues is unsurprising given the metabolic function and physiological role of these tissues in dehydration tolerance.

Contrasting results for significantly differentially abundant phosphopeptides in the two tissues supports a distinct liver response and higher variability in muscle. Liver showed a greater number of differentially abundant phosphopeptides (Figures 3A and 3B), the lack of which in muscle appears to be due to variation between muscle samples rather than a lack of identified phosphopeptides or distribution of average abundance within conditions (Figure 3C). Interestingly, although there is significant overlap between all proteins identified with corresponding phosphopeptides in liver and muscle (Figure 4A), when we look at only proteins with significantly differentially abundant phosphopeptides the overlap decreases below what would otherwise be expected (Figure 4B). Rather than maintaining the same ratio of overlapping proteins, the overlap drops to where most liver proteins are not differentially abundant in the muscle, but 6 of 15 proteins in the muscle are also differentially abundant in the liver. This further suggests that not only is the liver displaying a liver-specific phosphorylation pattern in response to dehydration, but in so far as the muscle is mounting a response, it appears unlikely as a substantial subset of changing phosphopeptides in muscle also change in liver, possibly suggesting that these shared phosphopeptides represent a more global response to dehydration.

### Central Metabolic Pathways Enriched for Differentially Abundant Phosphopeptides

Functional analysis revealed enrichment of different processes in the liver and muscle. In the liver, central metabolism pathways are enriched for phosphoproteins including glycolytic/gluconeogenic pathways, the pentose phosphate cycle, biosynthesis of amino acids, and citrate metabolism (Figures 5 and 7). This aligns with previous research showing increased anaerobic metabolism (Hillman, 1978a; Katzenback et al., 2014) and mobilization of glucose into the plasma (Malik and Storey, 2009). Dehydration tolerance in this animal is thought to be limited by circulatory oxygen delivery such that as dehydration increases, hematocrit is elevated and resting heart rate increases to maintain oxygen delivery (Hillman, 1978b, 1978a). At higher levels of dehydration, oxygen delivery is no longer sufficient and anaerobic glycolysis increases as seen by elevated whole-body lactate levels (Hillman, 1978a).

Similarly, biosynthesis of amino acids in the liver (Figure 7) correlates with previous observations of increased plasma and liver free amino acid levels in laboratory dehydrated and naturally estivating *X. laevis*, the opposite results seen in starvation alone (Balinsky et al., 1967; Unsworth and Crook, 1967). The physiological relevance of increased amino acid levels is not clear, although given that the increase is not uniform across all amino acids it may be a sum of different pathways such as the urea cycle, TCA cycle, and other liver functions involving specific amino acids (Balinsky et al., 1967). This corroboration shows that gene set analysis in the liver closely predicts the physiological and metabolic adaptations of dehydration tolerance that have thus far been determined in *X. laevis*.

### Structural Proteins Only Commonality between Liver and Skeletal Muscle

Gene set analysis also revealed pathways and processes that had not been previously implicated in *Xenopus* dehydration. Several actin and cytoskeletal-related processes and molecular functions were identified as being overrepresented for proteins with corresponding significantly differentially abundant phosphopeptides (Figures 5, S3 and S4). Although not previously identified as a dehydration tolerance adaptation in *X. laevis*, cytoskeletal reorganization is seen in other dehydration-tolerant species ranging from invertebrates to plants (Clark et al., 2009; Cruz DE Carvalho et al., 2014; Li et al., 2009). The loss of body water likely results in reduction in cell volume throughout the body, and thus cell morphology would be impacted, which could result in modification to the cytoskeleton to tolerate structural stresses.

Muscle tissue had few enriched terms, although like the liver they include cytoskeletal elements along with regulation of striated muscle contraction (Figure 6). Although the exact functional outcome of these terms being enriched has not been determined, potentially these are adaptations to the structural stresses encountered by cells losing cell volume given that dehydration of muscle tissue negatively impacts muscle contractile function (Lorenzo et al., 2019). It therefore is consistent with the physiology of muscle dehydration to find that regulation of striated muscle contraction was enriched in our dataset. Whether this is a result of dehydration or a response to dehydration is unknown; however, it can be speculated that regulation of this process by reversible phosphorylation could be an energy-saving mechanism while muscles are dormant, or perhaps a preparatory mechanism for when rehydration occurs.

### Regulators of Glycolysis Differentially Phosphorylated during Dehydration

Specific enzymes involved in anaerobic glycolysis show how this process may be regulated in response to dehydration. Pyruvate kinase is an ATP-producing, regulated step of glycolysis that shows increased activity in *X. laevis* liver during dehydration, potentially due to dephosphorylation (Dawson et al., 2018), which would support anaerobic glycolysis. Our analysis showed that pyruvate kinase was less phosphorylated during dehydration; however, this did not reach statistical significance (Table 1). LDH in the liver also shows altered kinetic and regulatory properties, potentially due to phosphorylation (Katzenback et al., 2014), although we did not identify LDH in our analysis. In this case, LDH is phosphorylated and has lower affinity for its substrates until physiological levels of urea are present, which restores kinetic parameters to control levels. The authors proposed that this is a preparatory mechanism for when rehydration occurs and urea is secreted, LDH may remain phosphorylated and therefore less active, shunting pyruvate into the TCA cycle (Katzenback et al., 2014).

The prominence of central metabolic pathways in our gene set enrichment analysis of the liver led us to examine which enzymes in these pathways exhibited differentially abundant phosphopeptides in response to dehydration. Particularly interesting was the identification of two PFKFB enzymes (Table 1). These bifunctional enzymes catalyze the conversion of Fru-6-P to Fru-2,6-P_2_ using a PFK2 domain, or the reverse using an FBP2 domain. Fru-2,6-P_2_ is the main allosteric activator of phosphofructokinase 1 (PFK1) that catalyzes the first committed ATP-utilizing step of glycolysis (the phosphorylation of fructose 6-phosphate to fructose 1,6-bisphosphate) therefore making PFKFB enzymes central regulators of glycolytic flux. Multiple PFKFB enzymes exist, PFKFB1 being the canonical liver isozyme that has previously been identified as a regulatory feature of dehydration tolerance in the wood frog (Hawkins et al., 2019). Like *X. laevis*, the wood frog survives severe dehydration, in part by funneling glycogenolytic end products through anaerobic glycolysis (Churchill and Storey, 1994). Wood frogs also survive whole-body freezing by converting the glycogenolytic end products to cryoprotective glucose and exporting it from the liver to the rest of the body. Both cases, carbon fluxing through glycolysis for energy or export as a cryoprotectant, appear to be controlled at the PFK1 locus and therefore regulated by PFKFB enzymes, potentially by phosphorylation of a serine homologous to human S33 (Hawkins et al., 2019; Vazquez Illanes and Storey, 1993).

Our results show phosphopeptides containing S31 and S32 from PFKFB1 (homologous to human PFKFB1 S33 and S34) are indeed decreased in the liver of *X. laevis* in response to dehydration (Table 1, Figure 8A). These residues are present in the PFK2 domain of this enzyme (Figure 8B) and when phosphorylated result in decreased PFK2 activity while increasing FBPase-2 activity (Kurland and Pilkis, 1995). Dephosphorylation of PFKFB1 during dehydration would therefore promote PFK2 activity and drive glycolysis. This result is consistent with previous studies showing increased anaerobic glycolysis and regulation of other glycolytic enzymes promoting glycolysis in *X. laevis* liver during dehydration (Dawson et al., 2018; Hillman, 1978a; Katzenback et al., 2014).

### Hypoxia-Inducible PFKFB3 Highly Upregulated and Phosphorylated

PKFB3 was also identified in our results, although this isozyme is not typically expressed in healthy liver tissue. The expression of PFKFB3 is prominent in a number of cancers (Shi et al., 2017) since the *pfkfb3* gene has multiple promoter HREs (binding site for HIF1α) and is thus induced under hypoxic conditions. This contributes to the high glycolytic rate of hypoxic tumor cells and has made PFKFB3 a target for pharmaceutical interventions (Hu et al., 2019; Mondal et al., 2019; Wang et al., 2018). Our results indicate that PFKFB3 is phosphorylated during dehydration at S463 (homologous to human S461) (Figure 8A), which is the residue that activates the PFK2 activity of this enzyme and therefore drives glycolytic flux (Bando et al., 2005). The mechanism of how phosphorylation of this residue increases PFK2 activity has not been studied; however, unlike in PFKFB1 the regulatory residue of PFKFB3 is in close proximity to the binding pocket of the FBPase-2 domain (Figure 8C). This could result in changes to the kinetics of this enzyme’s FBPase-2 activity rather than PFK2 activity, increasing the net conversion of Fru-6-P to Fru-2,6-P_2_ by inhibiting the reverse reaction. Phosphorylation of PFKFB3, as seen here, would likely contribute to anaerobic glycolysis during the proposed hypoxic conditions seen under high levels of dehydration in *X. laevis*.

Since the *pfkfb3* gene is known to be induced under hypoxic conditions, we measured the multiple transcript variants of this gene and found that all increased (∼4- to 10-fold) during dehydration (Figures 9A and 9B). As expected, this upregulation is specific to *pfkfb3* and not a general PFKFB response as the expression of *pfkfb1* actually decreased during dehydration (Figure 9C). This is likely a result of the eight HREs within the promoter of *pfkfb3* in *X. laevis*, more than the four found in the human *pfkfb3* promoter (Obach et al., 2004) and many more than the two found in the *X. laevis pfkfb1* promoter (Figure 9D). Together these results suggest a possible explanation for how anaerobic glycolysis is elevated in response to high levels of dehydration in *X. laevis*. High levels of dehydration restricts oxygen transport; this leads to a hypoxic cellular environment (Hillman, 1978a), which then stimulates transcription of *pfkfb3* (Figure 9B)*,* which is then activated by phosphorylation (Figure 8A), resulting in increased glycolytic flux as evident by elevated lactate levels (Hillman, 1978a).

## Conclusions

Here we present a phosphoproteomic analysis of *X. laevis* that shows tissue-specific responses to dehydration. Liver tissue exhibited a more defined pattern of phosphorylation and dephosphorylation compared with skeletal muscle that is likely the result of the metabolic function this tissue plays during dehydration exposure. Gene set analysis indicated that cellular functions and pathways related to glucose metabolism were highly enriched for differentially phosphorylated proteins in the liver, which led to the observation that PFKFB3, the hypoxia-inducible PFKFB isozyme, increased expression multiple fold and whose phosphopeptides were highly abundant during dehydration. Phosphorylation of PFKFB3 at S463 results in increased PFK2 activity of this enzyme, which could explain how anaerobic glycolytic flux increases in response to high levels of dehydration in *X. laevis*. Together, these results highlight the importance of the liver and expand our understanding of dehydration tolerance in this animal.

### Limitations of the Study

The lack of antibodies available for PFKFB isozymes in *X. laevis* means that protein levels and validation of these results by a second method will remain for future studies. How much PFKFB3 contributes to glycolytic flux will also need to be determined, either by the use of selective PFKFB isozyme inhibitors or fluxomic analysis, both of which present considerable technical challenges for live animal experiments. Furthermore, the dose-dependent effect of dehydration on PFKFB3 induction is unknown and leaves interesting questions to be explored.

### Resource Availability

#### Lead Contact

Kenneth B. Storey (kenstorey@cunet.carleton.ca).

#### Materials Availability

This study did not generate new unique reagents.

#### Data and Code Availability

The code generated during this study is available at: https://github.com/liamhawkins/thesis_phosphoproteomics_chapter.

## Methods

All methods can be found in the accompanying Transparent Methods supplemental file.

## Supplemental Information

Document S1. Transparent Methods, Figures S1–S4, and Table S1
