# Supplementary material for: Phosphoproteomic Analysis of Xenopus laevis Reveals Expression and Phosphorylation of Hypoxia-Inducible PFKFB3 during Dehydration
Source: iScience. 2020 Sep 22;23(10):101598. doi: 10.1016/j.isci.2020.101598 (PMC7554655; doi:10.1016/j.isci.2020.101598)
Supplement: Document S1. Transparent Methods, Figures S1–S4, and Table S1 [file mmc1.pdf]

iScience, Volume *23*

Supplemental Information

Phosphoproteomic Analysis of *Xenopus laevis* Reveals Expression and Phosphorylation of Hypoxia-Inducible PFKFB3 during Dehydration

Liam J. Hawkins, Xiaoshuang Wang, Xiaomin Xue, Hui Wang, and Kenneth B. Storey

Supplementary Table Titles

Table S1 Primer sequences for qPCR analysis. Related to **Figure 9**.

Supplementary Figure Titles

Figure S1 Summary of phosphopeptide from liver and muscle. **a)** Number of phosphopeptides containing phosphoserine (pS), phosphothreonine (pT), and phosphotyrosine (pY) residues. **b)** number of phosphopeptides containing one, two, or three phosphorylated residues. Related to **Figure 1**.

Figure S2 Hierarchical clustering of significantly differentially abundant phosphopeptides from liver and muscle of *Xenopus laevis* exposed to dehydration. Clustering of significantly differentially abundance phosphopeptides from **a)** liver and **b)** muscle. Related to **Figure 1**.

Figure S3 Semantic relation network of enriched GO cellular compartment terms in the liver of dehydrated *Xenopus laevis*. All other information as in **Figure 5.**

Figure S4 Semantic relation network of enriched GO molecular function terms in the liver of dehydrated *Xenopus laevis*. All other information as in **Figure 5.**

Table S1

Gene symbol	Accession/GeneID	Forward Primer	Reverse Primer	Product Size
pfkfb3.S all	431861	GTAGAGGGTTTCCCCACCAT	CTTTGCTGATCTCACAAGGGAAA	117
pfkfb3.S v2	XM_018255708	TGGATGCATTACTTCATGTTGCT	CCTGTGCTTCTCCCAGTCAA	115
pfkfb3.S v3	XM_018255709	CCAGCAGAAACTTGAACTGTGC	ACGGGTCAGCTTTTTGGAGAT	120
pfkfb3.S v4	XM_018255710	AGTAATCGAGCGACGGCTTT	GCGAACTAGCAGTACTCCCC	106
pfkfb1.S	NM_001095066	ATGCAGAATGAGGACAGGCTT	CGAGTCTGTTTGAGCTCTCG	119
sdha.L	NM_001090004	TCACGGCTTCTTAGCAGAGC	TGCTGAAGTGTCCTTCCGTC	120
atp5f1b.L	XM_018247436	TGCCCGTTTGTCTTGGGTAA	AACAGCCACACAAGTTGGTC	83

Figure S1

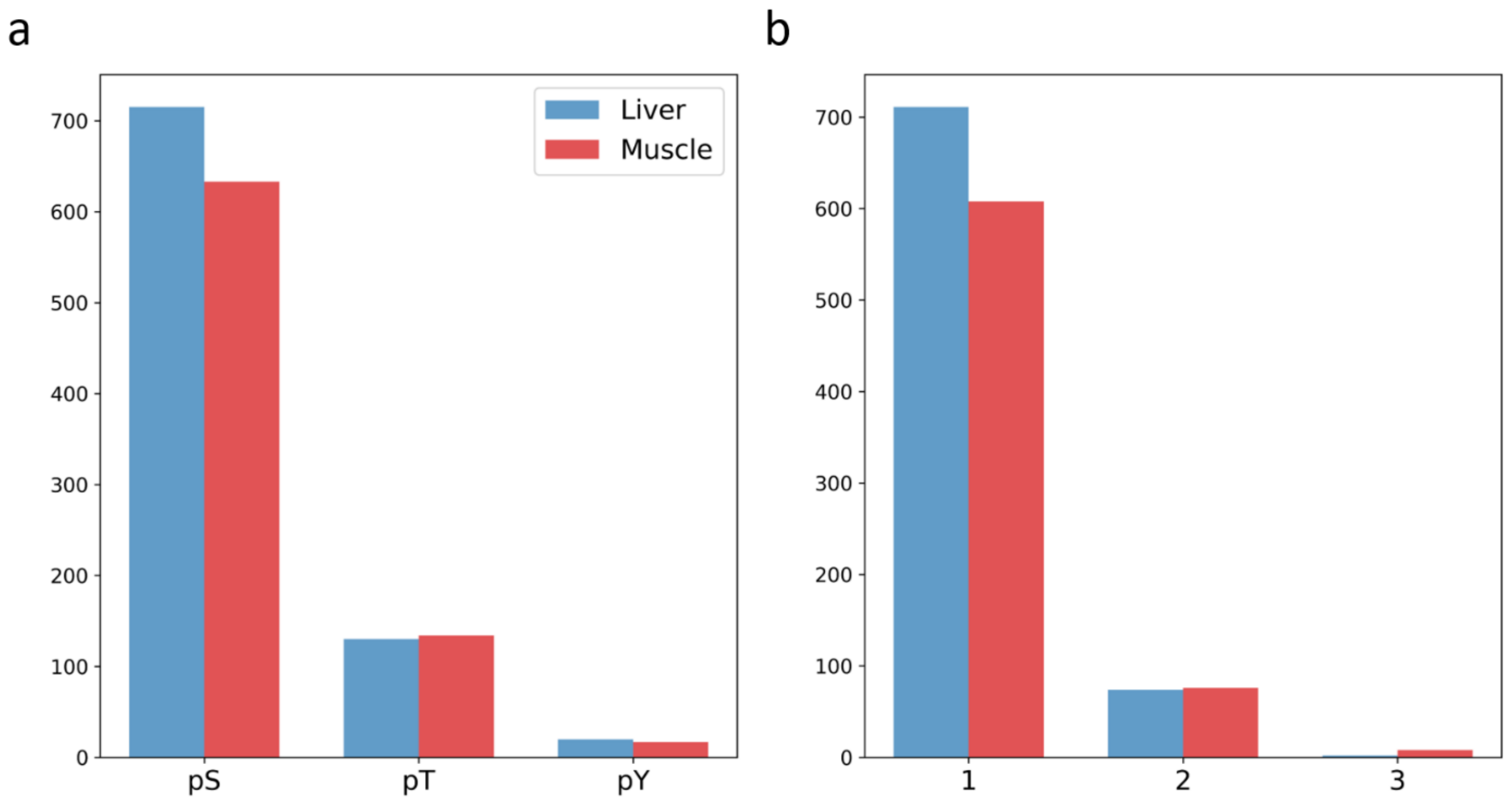

a
b
Liver
Muscle
700
600
500
400
300
200
100
0
pS
pT
pY
700
600
500
400
300
200
100
0
1
2
3

Figure S2

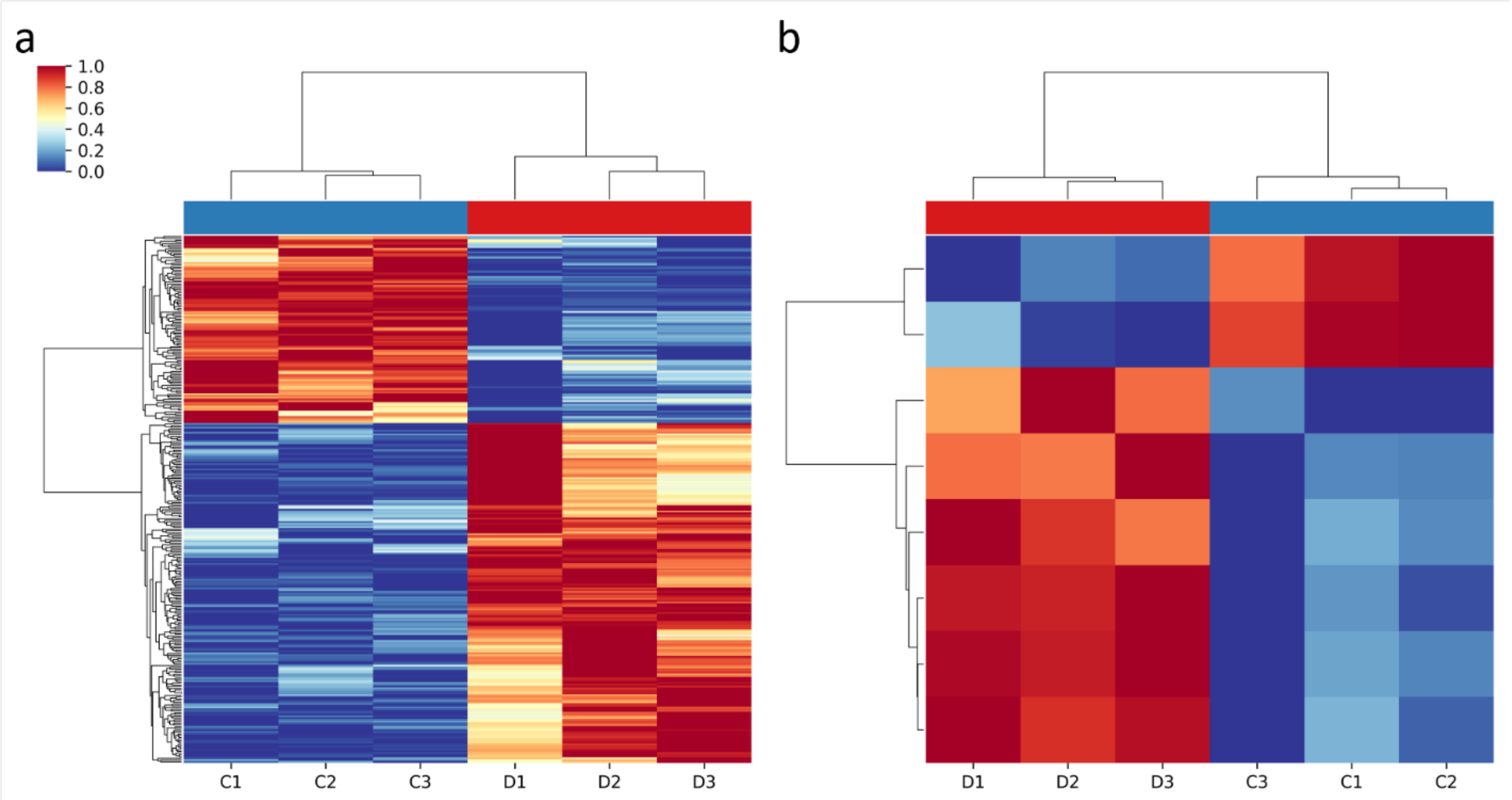

Figure S3

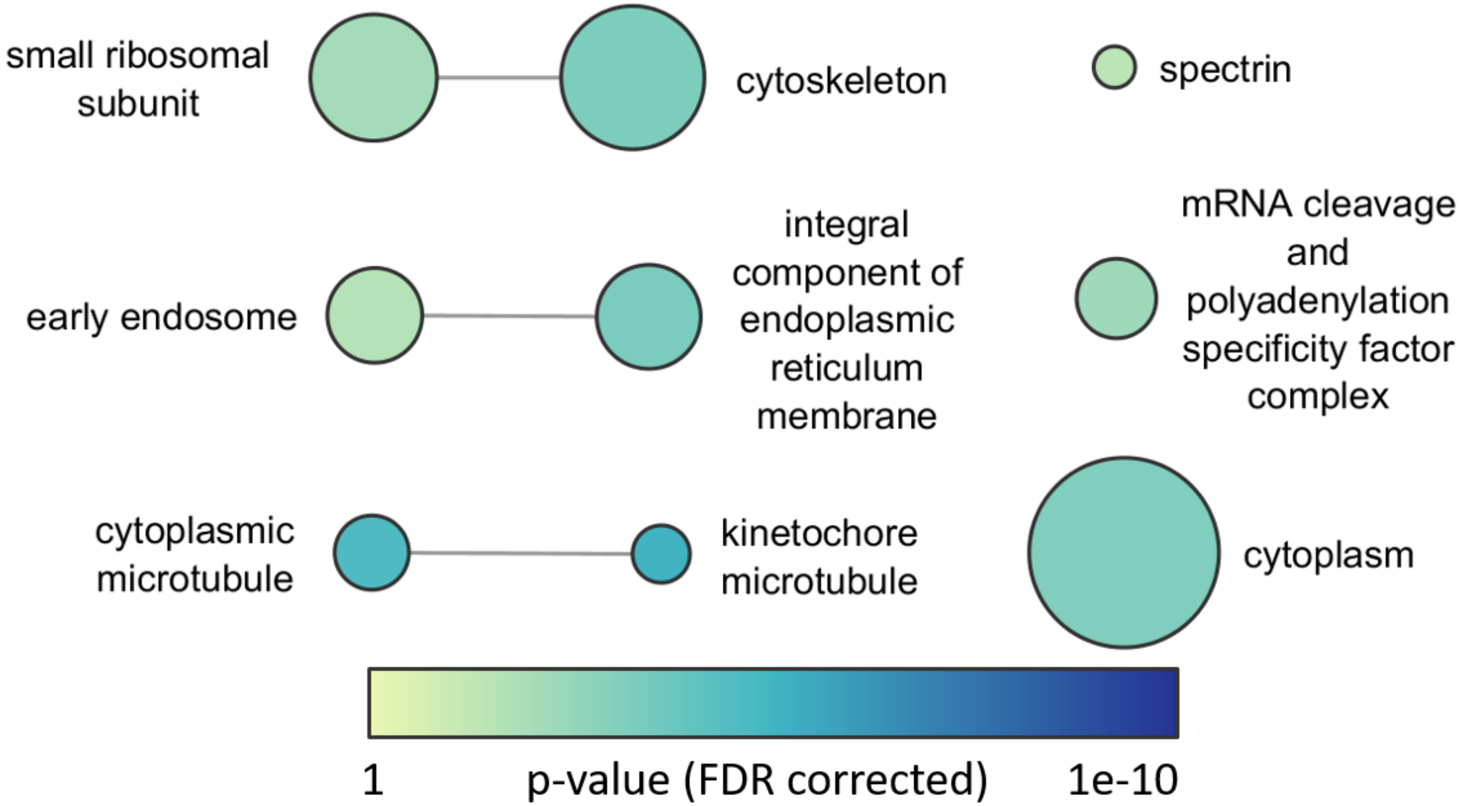
small ribosomal subunit
cytoskeleton
spectrin
early endosome
integral component of endoplasmic reticulum membrane
mRNA cleavage and polyadenylation specificity factor complex
cytoplasmic microtubule
kinetochore microtubule
cytoplasm
1
p-value (FDR corrected)
1e-10

Figure S4

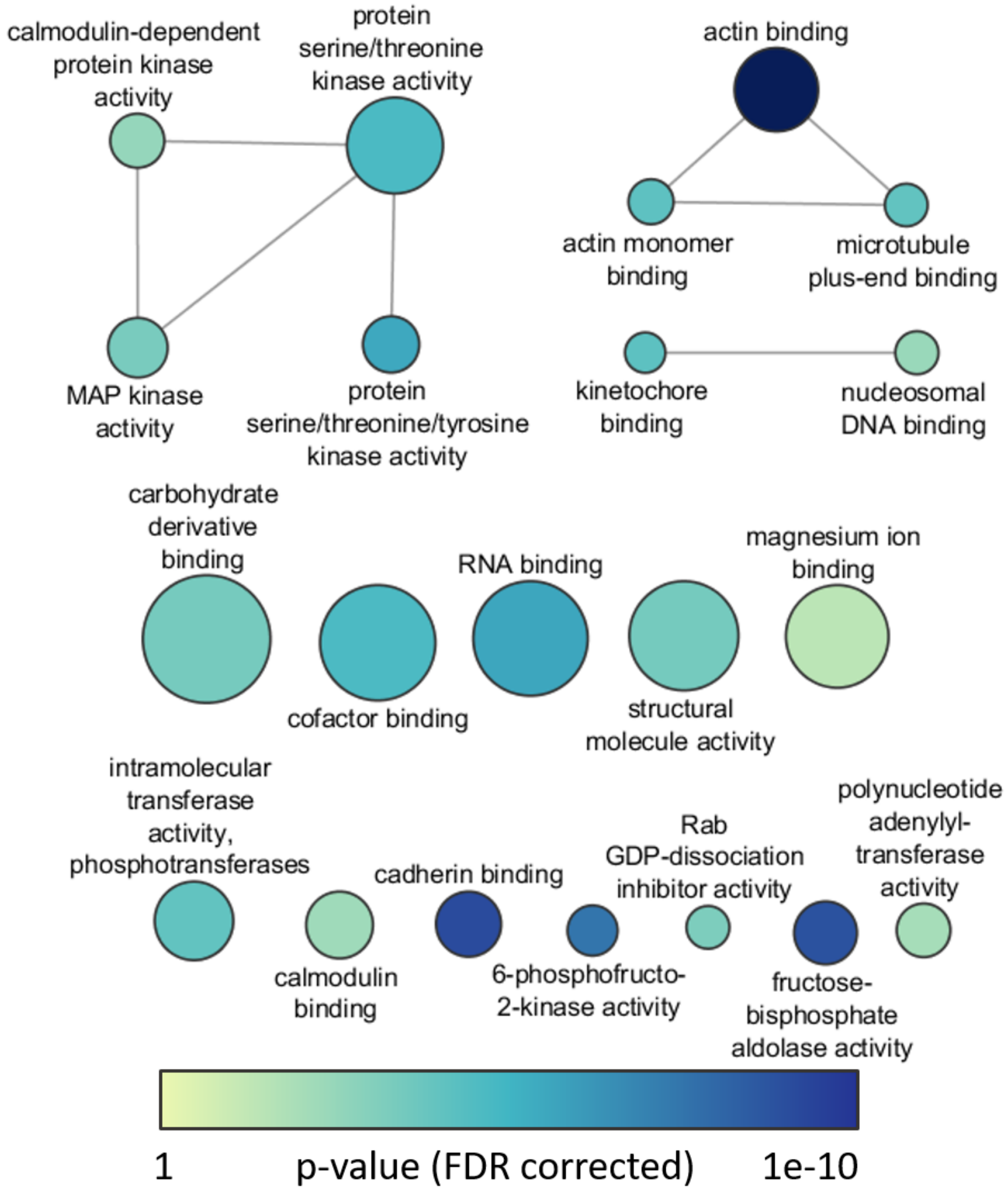
calmodulin-dependent protein kinase activity
protein serine/threonine kinase activity
actin binding
actin monomer binding
microtubule plus-end binding
MAP kinase activity
protein serine/threonine/tyrosine kinase activity
kinetochore binding
nucleosomal DNA binding
carbohydrate derivative binding
RNA binding
magnesium ion binding
cofactor binding
structural molecule activity
intramolecular transferase activity, phosphotransferases
cadherin binding
Rab GDP-dissociation inhibitor activity
polynucleotide adenylyl-transferase activity
calmodulin binding
6-phosphofructo-2-kinase activity
fructose-bisphosphate aldolase activity
1
p-value (FDR corrected)
1e-10

Transparent Methods

Animal experiments

Adult male *X. laevis* from the University of Alberta Science Animal Support Services were shipped to Carleton University where they were acclimated for one week at 22°C in aerated tanks filled with dechloraminated water. *X. laevis* were fed three times per week and the tank water was changed each feeding day. The animals were then randomly divided into control and dehydration exposure groups (mean body mass 64.40 ± 10.77 g and 64.58 ± 10.78 g respectively). Control animals were maintained in the above conditions. Animals in the dehydration exposure group were placed in dry tanks at 22°C and weighed periodically over the course of two days until they had lost approximately 30% body water (actual % body water loss 33.59 ± 2.58). Percent body water loss was calculated using the following equation:

$$\%\ Body\ water\ loss = \frac{m_i - m_d}{m_i \times BWC_i} \times 100\%$$

where m_i is the initial mass of the animal, m_d is the dehydrated mass, and BWC_i is initial body water content as previously determined (Malik and Storey, 2009). Control and dehydration exposed animals were then euthanized by pithing and skeletal muscle and liver tissues were quickly dissected, frozen in liquid nitrogen, and stored at -80°C until use. All animal protocols were approved by the Carleton University Animal Care Committee (protocol #106936) and conformed with the guidelines of the Canadian Council on Animal Care.

Protein extraction

Frozen skeletal muscle and liver tissue samples weighing 200 mg (n = 3 from each experimental condition) were extracted separately using EMD Millipore Cell Lysis Buffer (catalogue #43-040) with 1 mM EDTA, 1 mM EGTA, phosphatase inhibitors (1 mM Na_3VO_4, 10

mM NaF, 10 mM β-glycerophosphate) and protease inhibitors (BioShop catalogue #PIC001). Tissues were homogenized with a Dounce homogenizer in 1:4 w:v lysis buffer and incubated for 30 min on ice with intermittent vortexing. Total soluble protein lysates were then collected by centrifuging samples at 14,000 x g for 20 min at 4°C and taking the supernatant. Protein concentrations were determined using Bradford assay (Bio-Rad Cat #500-0005), then samples were frozen and lyophilized for 12 h using a Labconco FreeZone 12L freeze dryer and stored at room temperature until use.

Trypsin Digestion

For the following steps, samples were run in technical duplicate. Samples were reduced with 10 mM dithiothreitol for 30 min at 37°C then alkylated with 20 mM iodoacetamide for 30 min at 25°C in the dark with constant shaking. Samples were then trypsin digested (1:20 w/w, Promega, USA) for 12 h at 37°C with enzyme supplementation after 6 h and digestion efficiency monitored by LC-MS. The peptides were then desalted using a C18 column (CNW, China), according to manufacturer's directions and peptide samples were then normalized to a common concentration value using a BCA Protein Assay Kit (Pierce Biotechnology, USA).

Phosphopeptide enrichment

Phosphopeptides enrichment was performed using 10 mg aliquots of TiO_2 beads (GL Sciences, Japan). The beads were washed three times with 1 ml of loading buffer [65% acetonitrile (ACN), 2% trifluoroacetic acid (TFA), saturated by glutamic acid], then resuspended in 400 µl of loading buffer. A 2 mg aliquot of peptides were added to the beads and were incubated for 1 h at room temperature with constant shaking. The beads were then washed with wash buffer A (65% ACN + 0.5% TFA) and twice with wash buffer B (65% ACN + 0.1% TFA). Phosphopeptides were eluted with 200 µl of elution buffer A (0.3 M NH4OH + 50% ACN) and

two times with 200 µl of elution buffer B (0.5 M NH4OH + 60% ACN), each with constant shaking for 20 min at 20°C.

Phosphopeptide samples were separated by high-pH reverse phase high performance liquid chromatography (RP-HPLC) using a Waters e2695 Separation Module with a Durashel-C18 column (5 µm particle size, 100 Å pore size, 4.6 mm x 250 mm, Agela) with solvent A (5 mM ammonium formate, pH 10.0) and solvent B (ACN with 5 mM ammonium formate, pH 10.0). The liquid phase separation linear gradient was 5% solvent B for 15 min, followed by 5-50% solvent B for 70 min with a flow rate of 1 ml/min. Sixty fractions were collected in 1 min intervals and were then combined into six groups of 10 that were lyophilized and stored at -80°C until use.

Spectral library construction and analysis

Samples were resuspended in 0.1% formic acid and 2% ACN. Indexed Retention Time (iRT, Biognosys) reagent was added to each sample to calibrate retention times between respective elution fractions of each original sample. Each elution was then analysed using a nanoACQUITY UPLC M-Class system (Waters, USA) and a Q Exactive HF mass spectrometer (Thermo Fisher Scientific, USA). Samples were loaded onto a C18 trap column (5 µm particle size, 100 Å pore size, 180 µm x 20 mm; Waters, USA) followed by a C18 analytical column (1.8 µm particle size, 101 Å pore size, 100 µm x 150 mm; Waters, USA) at a flow rate of 300 nl/min using a linear 2-8% gradient of solvent B for 6 min, then a 8-35% solvent B gradient for 106 min, and finally a 35%-90% solvent B gradient for 13 min. Each sample was then loaded using electrospray ionization with spray voltage set to 2.0 kV and capillary temperature set to 290°C with the following parameters: (a) scanning range was 350-1200 m/z, the resolution of precursor ions was set to 60,000, the automatic gain control target was 3x106, and the maximum ion injection time

was 50 ms; (b) high-energy collision dissociation was performed with a 27% normalized collision energy; (c) the resolution of MS2 was set to 15,000 m/z, automatic gain control was set to 1x105, maximum ion injection time was set to 45 ms, and dynamic exclusion time was set to 30 s; (d) spectra were recorded in profile mode.

Using Proteome Discoverer 2.2 (Thermo Fisher Scientific, USA), the DDA mass spectrometry results were searched to construct DDA spectral libraries. The results for the elution groups were combined for searching. The database was created from *X. laevis* protein sequences (56748 sequences, 2018/04/01) retrieved from UniProt. The search parameters were as follows: (a) trypsin digestion (full with two missed cleavages allowed), (b) precursor mass tolerance of 10 ppm, (c) fragment mass tolerance of 0.02 Da, (d) variable modifications included N-terminal acetylation, methionine oxidation, phosphorylation of serine, threonine, and tyrosine, (e) fixed modifications, carbamidomethylation of cysteine residues, (f) HCD fragmentation mode, and (g) FDR is set to 1%.

Data analysis and differential phosphorylation analysis

For each tissue, non-phosphorylated peptides and phosphopeptides not present in at least two control and two dehydrated biological replicates were filtered out of the dataset. For each phosphopeptide, $\log_2$-fold changes and standard deviations between control and dehydrated animals were calculated using the following equations:

$$\log_2 FC = \log_2 \frac{AVG\left(\begin{bmatrix} Deh_1 \\ \vdots \\ Deh_n \end{bmatrix}\right)}{AVG\left(\begin{bmatrix} Con_1 \\ \vdots \\ Con_n \end{bmatrix}\right)}$$

$$\log_2 SD = \sqrt{SD(\log_2 \begin{bmatrix} Con_1 \\ \vdots \\ Con_n \end{bmatrix})^2 + SD(\log_2 \begin{bmatrix} Deh_1 \\ \vdots \\ Deh_n \end{bmatrix})^2}$$

Student's *t*-test with a Benjamini-Hochberg multiple test correction was used to determine statistical significance (FDR corrected p-value < 0.05).

Hierarchical clustering and principle component analysis

Hierarchical clustering was performed on samples using the *seaborn* python package (Waskom et al., 2014) which uses the *scipy* python package (Jones et al., 2001). Samples were clustered based on the Euclidean distance between the relative quantities of their respective phosphopeptides and phosphopeptides were clustered based on the Euclidean distance between their relative quantity in each sample. Principle component analysis (PCA) of samples was performed using the *sklearn* python package (Pedregosa et al., 2011).

Gene set analysis

Overrepresentation analysis of gene ontology (GO) terms (The Gene Ontology Consortium, 2019) and Kyoto Encyclopedia of Genes and Genomes (KEGG) pathways (Kanehisa et al., 2019) was performed using the WebGestalt webserver (Liao et al., 2019). While WebGestalt does not have built-in support for *X. laevis*, custom GO and KEGG annotation databases can be created and used with this tool. First GO and KEGG annotations for all *X. laevis* proteins were retrieved from UniProt (UniProt Consortium, 2019) and GO and KEGG gene matrix transposed (GMT) files were created using a custom python script to be used as the functional database in WebGestalt. The proteins corresponding to significantly differentially expressed (FDR-corrected p-value < 0.05) phosphopeptides were uploaded as the gene list, all proteins with GO or KEGG

annotations were uploaded as the background reference list of genes and the Benjamini-Hochberg procedure was used as a multiple test correction.

Over represented GO terms were further explored using REVIGO (Supek et al., 2011) which produces network diagrams of semantic similarity between GO terms using the hierarchical graph structure of GO. Significantly enriched GO terms and their FDR-corrected *p*-values produced by WebGestalt were uploaded using the default REVIGO settings and network diagrams of these significantly enriched terms were retrieved.

RNA extraction and cDNA synthesis

For RNA extraction, 50 mg of tissue from each sample was homogenized using a Polytron homogenizer in 1 mL of TRIzol reagent (Invitrogen; Cat# 15596-018). A 200 µL volume of chloroform was added to each sample which was then centrifuged at 10,000 × g at 4°C for 15 min. The aqueous phase containing total RNA was removed and precipitated using 500 µL of 2-propanol for 10 min at room temperature, then pelleted at 10,000 × g at 4°C for 15 min. Each pellet was then washed twice with ethanol and air-dried before resuspension in 50 µL of RNase-free water. RNA concentration and purity were determined using a BioTek Take3 microspot plate and PowerWave HT microplate spectrophotometer where all samples had 260/280 ratios of ~2.0, then RNA integrity was verified using a 1% agarose gel.

Reverse transcription cDNA synthesis was performed on 2 µg of RNA from each sample in 10 µL of RNase-free water using the Invitrogen M-MLV Reverse Transcriptase Kit (Cat# 28025013). A 1 µL volume of 200 $ng \cdot \mu L^{-1}$ oligo(dT) (Sigma-Aldrich) was added to each sample and incubated at 65°C for 5 min. Samples were then cooled on ice and the reverse transcriptase reaction was performed according to the manufacturer's directions using 1 µL of 10 mM dNTPs (BioShop, Canada).

Primer design and RT-qPCR

Primers were designed using Primer-BLAST (https://www.ncbi.nlm.nih.gov/tools/primer-blast/) using default settings with the exception of a PCR product size of 80-120 bp, and primer pair specificity checking for *Xenopus laevis* (taxid: 8355). Primers were synthesized by Integrated DNA Technologies and sequences are shown in **Table S1**.

RT-qPCR was performed using a Bio-Rad CFX Connect as previously described (Pellissier et al., 2006). PCR reactions were comprised of 8 μL cDNA, 2 μL qPCR buffer (100 mM Tris-HCl [pH 8.5], 500 mM KCl, 1.5% v:v Triton X-100, and 20 mM $MgCl_2$), 0.16 μL of 25 mM dNTPs, 4 μL of 1 M trehalose, 0.5 μL of formamide, 0.025 μL of 100X SYBR Green in DMSO (Invitrogen; #S7585), 0.5 μL of 0.3 nmol·μL^{-1} of each primer, 0.125 μL of 5U·μL^{-1} wild-type Taq (BioShop; #TAQ001.1) and autoclaved ddH2O for a final volume of 20 μL. The following thermocycle series was used: An initial 95°C denaturing step for 3 min, then 40 cycles of denaturing at 95°C for 10 sec, annealing at 57°C for 20 sec, and extension at 72°C for 20 sec. A melting analysis was performed from 55-95°C to ensure the amplification of single products, and a 2-fold dilution standard curve for each target was run on quantification plates to ensure reaction efficiencies between 90-110%. Suitable reference genes in liver tissue were previously determined to be *atp5f1b.L* and *sdha.L* (Hawkins et al., 2018) and quantification was performed using Bio-Rad CFX Maestro 1.1 which employs the Pfaffl method (Pfaffl, 2001).

Data visualization

Unless otherwise stated figures were created using the *matplotlib* python package (Hunter, 2007) and SigmaPlot 12.5. Hierarchical clustering was visualized using the *seaborn* python package (Waskom et al., 2014) and visualization of REVIGO GO network diagram was done

using Cytoscape (Su et al., 2014). *De novo* protein modeling of *X. laevis* PFKFB1 and PFKFB3 was done using I-TASSER (Yang et al., 2015) and visualized using PyMOL (https://pymol.org).

References

Hawkins, L.J., Luu, B.E., Storey, K.B., 2018. Selection of reference genes for accurate RT-qPCR analysis of dehydration tolerance in *Xenopus laevis*. Gene Reports 13, 192–198. https://doi.org/10.1016/j.genrep.2018.10.006

Hawkins, L.J., Wang, M., Zhang, B., Xiao, Q., Wang, H., Storey, K.B., 2019. Glucose and urea metabolic enzymes are differentially phosphorylated during freezing, anoxia, and dehydration exposures in a freeze tolerant frog. Comp. Biochem. Physiol. Part D Genomics Proteomics 30, 1–13. https://doi.org/10.1016/j.cbd.2019.01.009

Hunter, J.D., 2007. Matplotlib: A 2D Graphics Environment. Comput. Sci. Eng. 9, 90–95. https://doi.org/10.1109/MCSE.2007.55

Jones, E., Oliphant, T., Peterson, P., 2001. SciPy: Open Source Scientific Tools for Python.

Kanehisa, M., Sato, Y., Furumichi, M., Morishima, K., Tanabe, M., 2019. New approach for understanding genome variations in KEGG. Nucleic Acids Res. 47, D590–D595. https://doi.org/10.1093/nar/gky962

Liao, Y., Wang, J., Jaehnig, E.J., Shi, Z., Zhang, B., 2019. WebGestalt 2019: gene set analysis toolkit with revamped UIs and APIs. Nucleic Acids Res. 47, W199–W205. https://doi.org/10.1093/nar/gkz401

Malik, A.I., Storey, K.B., 2009. Activation of extracellular signal-regulated kinases during dehydration in the African clawed frog, *Xenopus laevis*. J. Exp. Biol. 212, 2595–603.

Pedregosa, F., Varoquaux, G., Gramfort, A., Michel, V., Thirion, B., Grisel, O., Blondel, M., Prettenhofer, P., Weiss, R., Dubourg, V., Vanderplas, J., 2011. Scikit-learn: Machine learning in Python. J. Mach. Learn. Res. 12, 2825–2830.

Pellissier, F., Glogowski, C.M., Heinemann, S.F., Ballivet, M., Ossipow, V., 2006. Lab assembly of a low-cost, robust SYBR green buffer system for quantitative real-time polymerase chain reaction. Anal. Biochem. 350, 310–2. https://doi.org/10.1016/j.ab.2005.12.002

Pfaffl, M.W., 2001. A new mathematical model for relative quantification in real-time RT-PCR. Nucleic Acids Res. 29, e45. https://doi.org/10.1093/nar/29.9.e45

Su, G., Morris, J.H., Demchak, B., Bader, G.D., 2014. Biological network exploration with Cytoscape 3. Curr. Protoc. Bioinforma. 47, 8.13.1-24. https://doi.org/10.1002/0471250953.bi0813s47

Supek, F., Bošnjak, M., Škunca, N., Šmuc, T., 2011. REVIGO summarizes and visualizes long lists of gene ontology terms. PLoS One 6, e21800. https://doi.org/10.1371/journal.pone.0021800

The Gene Ontology Consortium, 2019. The Gene Ontology Resource: 20 years and still GOing strong. Nucleic Acids Res. 47, D330–D338. https://doi.org/10.1093/nar/gky1055

UniProt Consortium, 2019. UniProt: a worldwide hub of protein knowledge. Nucleic Acids Res. 47, D506–D515. https://doi.org/10.1093/nar/gky1049

Waskom, M., Botvinnik, O., Hobson, P., Warmenhoven, J., Cole, J.B., Halchenko, Y., Vanderplas, J., Hoyer, S., Villalba, S., Quintero, E., Miles, A., 2014. Seaborn: statistical data visualization. https://doi.org/10.5281/zenodo.1313201

Yang, J., Yan, R., Roy, A., Xu, D., Poisson, J., Zhang, Y., 2015. The I-TASSER Suite: protein structure and function prediction. Nat. Methods 12, 7–8. https://doi.org/10.1038/nmeth.3213
